# Effective Coverage for Antiretroviral Therapy in a Ugandan District with a Decentralized Model of Care

**DOI:** 10.1371/journal.pone.0069433

**Published:** 2013-07-23

**Authors:** Florian J. B. Scheibe, Peter Waiswa, Daniel Kadobera, Olaf Müller, Anna M. Ekström, Malabika Sarker, H. W. Florian Neuhann

**Affiliations:** 1 Institute of Public Health, University of Heidelberg, Heidelberg, Germany; 2 Department of Cardiology/Pulmonology, Ortenau Klinikum, Offenburg, Germany; 3 Department of Health Policy, Planning and Management, Makerere University School of Public Health, Kampala, Uganda; 4 Division of Global Health, IHCAR, Karolinska Institutet, Stockholm, Sweden; 5 Iganga/Mayuge Health and Demographic Surveillance Site, Iganga, Uganda; 6 James P. Grant School of Public Health, BRAC University, Dhaka, Bangladesh; Vanderbilt University, United States of America

## Abstract

**Introduction:**

While increasing access to antiretroviral therapy (ART) is reported from many African countries, data on effective coverage particular from settings without external support or research remains scarce. We examined and report effective coverage data from a public ART program in rural Uganda.

**Methods:**

We conducted a retrospective cohort study at all ART-providing governmental health facilities in Iganga District, Eastern Uganda. Based on all HIV patients registered between April 2004 and September 2009 (n = 4775), we assessed indicators of program performance and determined rates of retention and Cox proportional hazards for attrition. Effective ART coverage was calculated using projections (SPECTRUM software) adapted to the district demographic structure and number of people receiving ART.

**Results:**

By September 2009, district public sector effective ART coverage was 10.3% for adults and 1.9% for children. After a median follow-up of 26.9 months, overall ART retention was 54.7%. The probability of retention was 0.72 (95% confidence interval (CI) 0.69–0.75) at 12 and 0.58 (CI 0.54–0.62) at 36 months after ART initiation. Individual health facilities differed considerably regarding performance indicators and retention. Overall, 198 (16.9%) individual files of 1171 registered ART patients were lost. Young adult age (15–24 years) had a higher risk of attrition (HR 2.1, CI 1.4–3.2) as well as WHO stage I (HR 4.8, CI 1.9–11.8) and WHO stage IV (HR 2.5, CI 1.3–4.7). An interval ≥6 weeks between HIV testing and ART initiation was associated with a reduced risk (HR 0.6, CI 0.47–0.78).

**Conclusion:**

Compared to reported national data effective ART coverage in Iganga District was low. Intensified efforts to improve access, retention in care, and quality of documentation are urgently needed. Children and young adults require special attention in the program.

## Introduction

Over the last years, scaling up and decentralization of antiretroviral therapy (ART) programs have been achieved in many countries in Sub-Saharan Africa (SSA). The Joint United Nations Programme on HIV/AIDS (UNAIDS) reported an ART coverage of 47% for SSA in 2010. [1] However, limited district-level data are available assessing retention in care and coverage, in particular outside of research settings.

Uganda has about 32.9 million inhabitants [2] and faces a generalized HIV epidemic, with an estimated 1.2 million persons living with HIV (PLWH) and around 64,000 AIDS-related deaths per year. [3] Since ART became available through the public sector in 2003, the ART program under the Ugandan Ministry of Health is undergoing a scale-up process [4].

Efforts to further scale up access to ART are supported by recent research showing an important risk reduction of HIV transmission under ART and the consequently proposed strategy to use treatment as prevention. [5] This is also reflected by the new UNAIDS and WHO strategies aiming at universal access to ART by the year 2015. [6] However, as the scale-up process and expansion of ART coverage is going on, it appears that apart from still limited access to ART, the long-term retention under therapy also constitutes a major challenge in SSA countries. [7]–[9] Low retention rates give reason to re-evaluate the effective coverage of ART. High drop-out rates and a rising frequency of drug resistances are of increasing concern to sustain an effective response to the pandemic. [10]–[12] Recent findings show that almost one in four patients lost to follow up (LTFU) returning into care presents with drug-resistance to first line treatment [13].

For Uganda, a national ART coverage of 53.5% and a retention rate of over 80% at 24 months of ART have been reported. [14], [15] Previous studies evaluating ART programs in Uganda are from faith-based settings [16], NGO-based settings [17]–[20], prospective research cohorts [18]–[23], or without inclusion of peripheral health centres (HC) [18], [20], [24] and give an incomplete picture of ART in rural Uganda.

The objective of this study was to describe and analyze the evolution of scale-up and decentralization of ART, retention in care, as well as the resulting effective ART coverage in a rural district in Eastern Uganda.

## Methods

### Study Setting

This retrospective study was conducted in Iganga District, east central Uganda, with 682,100 inhabitants in 2009. [25] Apart from Iganga town the district is predominantly rural and the inhabitants are principally subsistence farmers with only few industries located in the district. [25], [26] Iganga town and a large share of Iganga District are part of the Iganga-Mayuge Health and Demographic Surveillance Site (HDSS). The Ugandan 2004/2005 sero-behavioral survey revealed a countrywide HIV prevalence of 6.4% (age-group 15–49 years) and a similar one for the east central Region (6.5%). [27] The 2011 HIV survey showed a slight increase of the national HIV prevalence with 7.3% [95% CI 6.8% to 7.9%] in the same age group with rates for the east-central region remaining stable at 5,8% [95% CI 4.2% to 7.5%] (6.7% among women and 4.8% among men). [28], [29] A direct comparison of the data is however limited since the regional boundaries were changed between the 2 surveys.

Health facilities in the district comprise one public general hospital in Iganga town, three health centres (HC) level IV and 9 HC level III. [30] A HC III targets a population of about 20,000 inhabitants, provides inpatient and outpatient care and is officially run by one clinical officer, one nurse, two midwives, one nursing assistant and assisting staff. [31] HCs level IV serve the health sub-district and target about 100,000 people each. They provide all the services of HC III plus surgery and should have at least one physician, two clinical officers, two nurses, two midwifes, two nursing assistants and several other assistants for laboratory or dispensing [31].

As from April 2004, ART was introduced at the hospital followed by a gradually decentralization of the ART program with one rural HC level IV providing ART since December 2005 and three others since April/May 2007, out of them one HC level III. This HC III hosts a research station for trypanosomiasis and thus had better staffing and laboratory equipment than a normal HC III which was the reason why it became an ART site. It’s infrastructure and staff involved in ART care however did not differ from the three HC level IV under investigation.

Despite the allocation of a medical officer, during 18 visits to HCs level IV during ART clinic days, no physician was found to be present. At the HCs level IV ART clinic tasks were shifted to either Clinical Officers, nurses or midwifes who had all undergone a standardized national training course for comprehensive HIV care and ART. At the HC level III a trained Clinical officer and a trained nurse were in charge of the ART program. Likewise at the hospital, nurses were in charge of the ART clinics, although here physicians were available for consultations. No HC had an electronic data system for the ART clinic. All facilities included in the study provided HIV testing but no CD4 count was available in the district. Few patients could afford to pay for a CD4 count from a nearby mission hospital outside the district. Since 2009, a mobile quality control team from the district health office provides ongoing support and monitoring on a regular basis but no pediatric advice for ART was available by the time of our study.

ART clinics in Iganga District operated once or twice a week and patients got a supply of antiretrovirals (ARVs) for 30 days. Per facility, one ART-register and individual HIV files for each patient are used for documentation. Every patient is routinely weighed and in case of clinical symptoms seen by a clinical officer before drug dispensing. At all facilities, people living with HIV were assisting the health workers with weighing and file retrieval during ART clinic days.

At the hospital drug supply was partly funded via the DELIVER project [32] and supplied by the Joint Medical Store [33]. The main part of ARVs at the hospital as well as all ARV supply for the HCs was delivered by the National Medical Store [34] under the Ministry of Health. Drugs had to be ordered by the in-charge of the ART program at the facilities via fax order forms two months prior and were distributed via the district health office.

### ART Eligibility and Regimens

During the study period, Ugandan national guidelines set ART eligibility for adults and adolescents (≥15 years old) at a CD4 threshold below 200 (until 2008) or 250/µl thereafter or, in absence of CD4 cell count, at clinical stage WHO III and IV. Since March 2008, the recommended first line regimen replaced stavudine for zidovudine, in combination with lamivudine and nevirapine or efavirenz [35].

### Population Estimates and HIV Projections

Using HDSS sentinel data and population projections for the district [25], we constructed an age pyramid of the district population for mid-year 2009.

We used SPECTRUM [computer program] Version 4.43 beta 7 with default parameters for Uganda and eligibility criteria for ART according to national guidelines to project the number of PLWH and the need for ART in Uganda. [36]–[38] As no HIV data exists specifically for Iganga District and as the 2004/2005 survey showed similar results for total Uganda and the Eastern Region [27], we applied the national SPECTRUM data to the district demographic structure to project the local number of PLWH and of those in need of ART.

### Data Collection

For this observational study, we extracted data retrospectively at health facility level using an audit of paper based patient files and HIV registers. [39], [40] The file audit tool included the following variables: patient demographics; date of HIV test; date of ART start; most recent documented WHO stage, last documented visit, but no personal identifier. Death and transfer-out to another health provider were not systematically recorded in the patient files but could be assessed at some facilities using the archive system.

For identification of the facility we used the labels “hospital” and HC “A”, “B”, “C”, and “D”. All patients diagnosed with HIV and registered between 1^st^ April 2004 and 1^st^ September 2009 were included. After piloting of the tool, the audit was performed in September 2009.

The completeness of the audit was ascertained in different ways: The purpose of our audit and the importance of getting access to every available patient file were explained to the in-charges at the five health facilities. Whenever the number of files present differed from the number of registrations, the in-charges were contacted again and asked to have a second look for remaining files. Physical search for files included all possible cabinets and drawers. The age distribution for the 32 patients without documented age was assumed to be the same as in the overall cohort for the calculation of coverage.

### Definitions

Every patient not attending the facility at least once during the 90 days prior to our review was considered to be no longer active in care and counted as attrition, unless the case could be identified as a transfer-out to another facility. Attritions were subtracted from patients retained in care. The *time point* of attrition was set at 30 days after the last documented visit. By this time, the last drug supply would have been consumed. The number of attritions was expressed as proportion per 100 person years of ART.

The effective coverage was expressed as the ratio of the number of patients currently receiving ART over the number of PLWH in need of ART [41].

### Data Analysis

**Figure 1:**
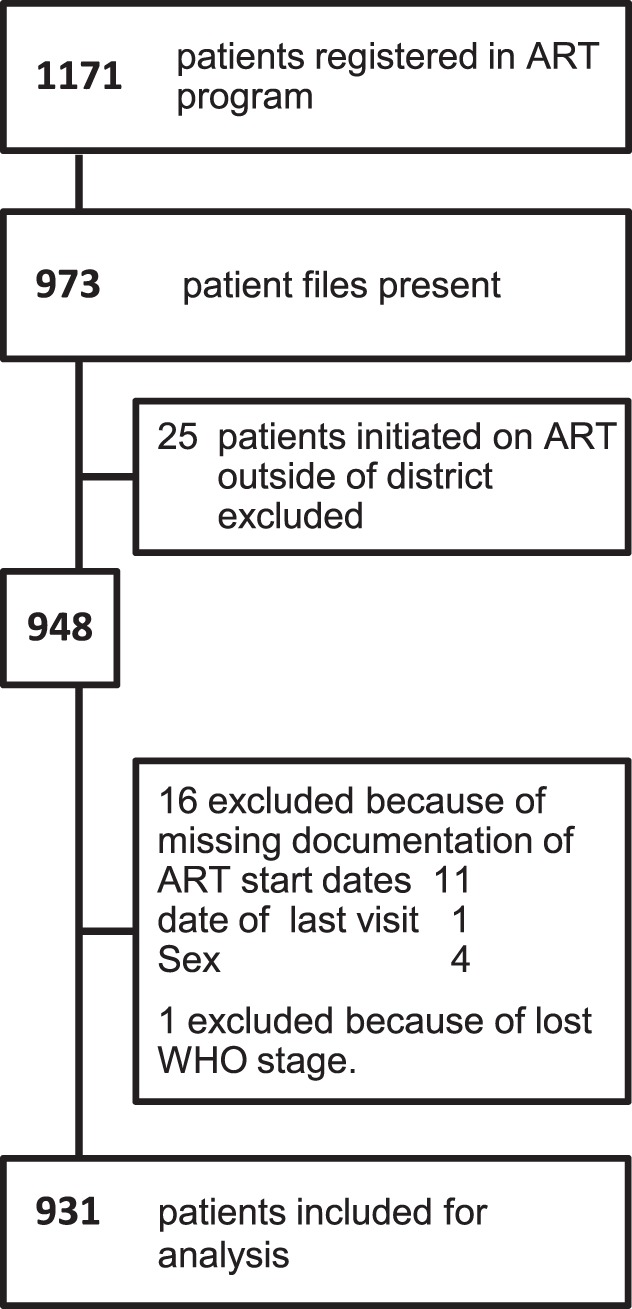
Patients included for hazard ratio analysis.

Data were captured in ACCESS and analyzed with STATA IC version 11.0. Confidence intervals (CI) were set at 95% and significance level at p≤0.05. Simple descriptive statistics included proportions, medians and inter-quartile ranges (IQR). Pearson’s Chi^2^ Test and Wilcoxon rank-sum test were used as required. Patient files with missing or incomplete documentation were excluded from further analysis (figure 1). Kaplan Meier estimate was used to calculate the probability of retention in care and Cox Hazard Ratios (HR) were used to identify factors associated with attrition. All factors significant in the univariate analysis were included in the multivariate model with a significance level set at p≤0.05.

### Ethical Approval

For this study we used information from routinely collected and documented data at the clinics from secondary sources like registers and reports. When using patients files as source, the data extraction form did not include personal identifiers. No additional primary data was captured and no direct researcher-patient interaction occurred for this study. Therefore individual informed consent was not required. The health district and all heads of the facilities were informed and agreed to the assessment. Files were only accessible at the study sites to the study personnel who kept information confidential. All data was kept in a secure database. The protocol for this analysis did not include an individual consent form, because personal consent by patients was not regarded as necessary nor even feasible in the absence of patients, patients lost to follow up etc, but we did not request an explicit waiver for consenting. Ethical approval for the use of routine clinic data in Germany depends entirely on the votum of the IRB. This study received approval from the Institutional Review Boards of the Makerere University Kampala, Uganda and the University Clinic Heidelberg, Germany.

## Results

Between April 2004 and 1^st^ September 2009, 4775 patients had been registered in the Iganga District HIV program and 1171 (24.5%) patients had been started on ART, contributing to a total of 2462 person-years with a median follow-up time of 26.9 months since ART initiation.

### Program Evolution

ART program characteristics are described in table 1. From program start to end of January 2005, only 8 patients had been initiated on ART at the hospital and nobody at HC level. February 2005 marks the beginning of a scale-up process at the hospital. Two years later, the decentralization to HC level began: By end of August 2007, 51 (9.1%) out of 558 ART patients ever enrolled were initiated at HC level. One year later, this proportion had increased up to 21.4% (184/860) and to 28.4% (333/1171) by the end of August 2009. The contribution to decentralized access varied between individual HCs: By the time of our study, HC C had enrolled more than twice the number of patients on ART than HC B after the same time span (table 1). Some facilities had higher rates of ART initiations per registered HIV patients than others (table 1).

**Table 1:** ART program characteristics by health facility in Iganga District, Uganda.

Health Center/facility	HC A	HC B	HC C	HC D	Hospital	Total
Duration of ART program in months by September 2009	45	29	29	28	65	–
Patients ever registered in Pre-ART care	939	642	509	267	2418	4775
Patients started on ART [I0] (% of ever registered)	125 (13.1%)	39 (6.1%)	96 (18.9%)	73 (27.3%)	838 (34.7%)	1171 (24.5%)
ART patient files present (% of ever started)	113 (90.4%)	21 (53.8%)	96 (100%)	41 (56.2%)	702 (83.8%)	973 (83.1%)
Documented Transfer-outs [T]	0	0	0	0	64	64
ART patients alive and attending* [AL]	85	18	78	16	380	577
Total attritions to ART program [AT]†	40	21	18	57	394	530
Retention in Care [%RT]‡	68.0%	46.2%	81.3%	21.9%	53.0%	54.7%
Attrition rate per 100 person years	23.5	48.3	18.2	58	19.2	21.5
Recognized and documented attritions	45.0%	4.7%	50.0%	0.0%	57.6%	48.1%
Documentation of essential data in patient files§	96.5%	81.0%	94.8%	36.6%	93.0%	91.0%

HC: Health Center;

* without transfer-outs;

† AT = I0 - AL - T;

‡ %RT = (AL+T)/I0;

§ age or year of birth, WHO stage and HIV test date documented in the file.

### Retention in Care, File Retrieval, and Documentation

A total of 973 ART patient files were present at the facilities corresponding to 83.1% of the 1171 documented ART starts according to registers (table 1). The number of lost files varied between the different facilities. HCs A and C and the hospital had more than 80% of the expected number of patient files present, whereas HCs B and D had less than 60% of the expected number of files present (table 1).

Out of the 973 patients with a retrieved ART file, 577 were still alive and attending and not transferred-out to other providers (table 1). In 64 cases, transfer-out could be ascertained resulting in an overall retention in care of 54.7% (641/1171) (table 1).

Site specific rates of retention in care varied considerably: While HCs A and C and the hospital had similar results for attrition rates per 100 person-years of observation, HCs B and D had much higher attrition rates (table 1). At the three other facilities (HC A, HC C and the hospital), about half of the attritions had been identified as LTFU, contrasting to 4.7% identified attritions in HC B and 0% in HC D (table 1). The completeness of documentation of essential patient data (including age or year of birth, WHO stage and HIV test date) also differed with HC B and D having the lowest rates (table 1).

The hospital was the only facility where patient files had been classified differentiating not only active patients or patients LTFU but also identifying 64 (7.6%) patients transferred-out. Transfer-outs were not documented at HC level. Among the 380 attritions at the hospital, 126 (33.2%) were classified LTFU and 101 (26.6%) registered as dead while 107 (28.2%) were not yet identified as being LTFU or dead. AT HC level, staff did not discriminate between the outcomes dead and LTFU.

### ART Patient’s Characteristics

The median age of patients on ART was 36 years [IQR 30–43 years] and few children (5.45%) or young adults (15–24 years) (4.61%) were among the patients with present files (table 2). Overall female predominance was slightly higher at HC level (67.9%) compared to the hospital (61.1%) and women were significantly younger at HC level (table 2). In 62 patient files (6.38%) no age or year of birth was documented. The distribution of WHO stages among registered cases also varied widely with a higher proportion of stage III diagnosis at the hospital (85.6%) than at HC level (85.6% vs. 48.0%, p<0.001) (table 2).

**Table 2:** ART patient characteristics.

			Hospital		Health Centres		Total District		p*
Total			702		271		973		
female		(%)	429	(61.1%)	184	(67.9%)	613	(63.0%)	.011
children <15 years		(%)	39	(5.6%)	14	(5.2%)	53	(5.5%)	.810
young adults 15–24 years		(%)	33	(4.9%)	9	(3.8%)	42	(4.6%)	.498
Age [median]	male	(IQR)	39	(33–45.5)	37	(32–46.5)	39	(32–46)	.825
	female	(IQR)	35	(30–42)	33.5	(28–40)	36	(30–41)	.047
	total	(IQR)	37	(31–43)	35	(30–42)	36	(30–43)	.032
WHO stage	I or II	(%)	26	(3.7%)	73	(26.9%)	99	(10.2%)	<.001
	III	(%)	601	(85.6%)	130	(48.0%)	731	(75.1%)	
	IV	(%)	62	(8.8%)	65	(24.0%)	127	(13.1%)	
	missing	(%)	13	(1.9%)	3	(1.1%)	16	(1.6%)	
Time from test to ART start in months [median]		(IQR)	4.3	(1.3–13.8)	4.9	(1.6–14.7)	4.5	(1.4–14.5)	.805
Time from ART start to review in days [median]		(IQR)	950	(455–1365)	433	(253–673)	719.5	(357–1193)	<.001

IQR: Inter Quartile Range;

* Pearson-Chi^2^ Test or Mann Whitney Test comparing Hospital to Health Centre results.

### Probability of Retention in Care

**Figure 2:**
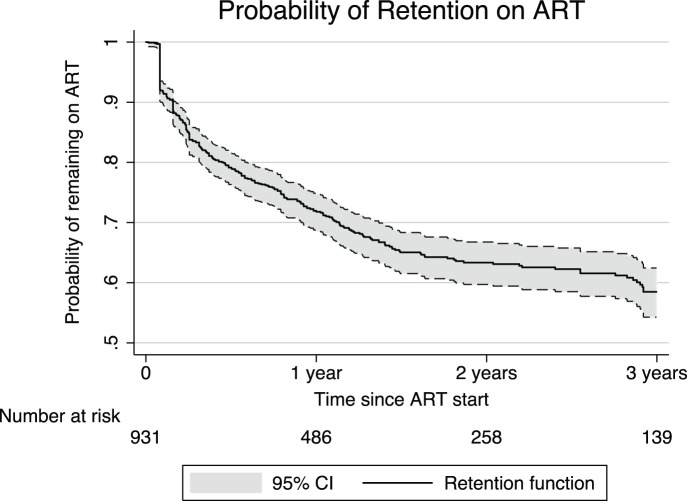
Probability of retention in care for ART patients in Iganga District, Uganda.

The probability of remaining on ART was 0.79 (95% CI 0.76–0.82) at 6 months, 0.72 (95% CI 0.69–0.75) at 12 months, 0.67 (95% CI 0.64–0.70) at 18 months, 0.63 (95% CI 0.60–0.67) at 24 months and 0.58 (95% CI 0.54–0.62) at 36 months after ART initiation (figure 2). In a sensitivity analysis including only health facilities having at least 80% of patient files present (hospital, HC A and HC C) we found similar rates of retention in care: 0.72 (95% CI 0.69–0.75) at 12 months and 0.60 (95% CI 0.55–0.64) at 36 months after ART initiation.

### Factors Associated with Attrition

Being a young adult at time of ART initiation, being in clinically staged “WHO I” or “WHO IV” and receiving ART at hospital level were factors associated with a significantly higher risk for attrition in multivariate analysis. In contrast, a period of at least 6 weeks between HIV testing and ART initiation was associated with a significant risk reduction for attrition (table 3).

**Table 3:** Adjusted hazard ratios for attrition to the ART program (n = 931).

Factor		Attrition to the program				HR (95% CI)	p value
		yes (%)		no (%)			
Sex							
female		187	(32%)	401	(68%)	0.80 (0.64–1.0)	0.061
male		130	(38%)	213	(62%)	1	–
Age							
<15 years		15	(30%)	35	(70%)	0.85 (0.50–1.5)	0.556
15–24 years		25	(60%)	17	(40%)	2.2 (1.5–3.3)	<0.01
≥25 years		248	(32%)	532	(68%)	1	–
missing		29	(49%)	30	(51%)	2.3 (1.3–3.9)	<0.01
WHO staging							
I		8	(42%)	11	(58%)	2.4 (1.0–5.7)	0.049
II		15	(19%)	63	(81%)	1	–
III		228	(33%)	470	(67%)	1.0 (0.58–1.7)	0.981
IV		58	(48%)	63	(52%)	2.4 (1.3–4.2)	<0.01
missing		8	(53%)	7	(47%)	3.1 (1.3–7.6)	0.012
Time from Test to ART start							
<6 weeks		101	(48%)	108	(52%)	1	–
≥6 weeks		180	(28%)	458	(72%)	0.59 (0.46–0.75)	<0.01
missing		36	(43%)	48	(57%)	0.57 (0.34–0.95)	0.032
Care level							
Health Center		69	(27%)	191	(73%)	1	–
Hospital		248	(37%)	423	(63%)	1.6 (1.2–2.2)	<0.01

HR: Hazard Ratio; CI: Confidence Interval; Each factor is adjusted for all other factors in the column.

### Coverage Estimate

Using the SPECTRUM software and the Iganga District population profile, we calculated a total number of 18,975 adult PLWH (≥15 years) and 4,246 children (<15 years) living with HIV in the district by midyear 2009 (see Table S1 Iganga District HIV Projections for 2009). Out of these PLWH, 5,855 (30.9%) adults and 2030 (47.8%) children were predicted to be in need of ART according to national guidelines. Therefore, the total number of PLWH in need of ART was 7,885 for midyear 2009. Applying a CD4 threshold for eligibility of 350 cells/mm^3^ for adults in 2009, would result in 9,661 PLWH in need of ART for that year. Using the predicted need for ART according to national guidelines (7,885), ART coverage in Iganga District according to registered treatment initiations was 14.9% (1171/7,885) in September 2009. Effective coverage according to patients retained in care was 8.1% (641/7,885) for all ages, 10.3% (602/5855) for adults and 1.9% (38/2030) for children.

## Discussion

Using all accessible data this study revealed a low rate of retention in ART care and an effective coverage rate much lower than expected when considering the reported national data.

Based on the number of patients active at the clinic by the time of our study i.e in September 2009 the district overall retention rate was 54.7% at a median follow-up time of 26.9 months which is much lower than the average of 76.8% at 26.3 months of follow-up in a meta-analysis of 39 cohorts from SSA. [7] We assume that 20–60% of the patients LTFU may have died as described before [42]–[44].

The relatively slow pace of scale up together with the low retention resulted in only 641 patients actively on ART by September 2009 which is substantially lower than the previously reported 1000 PLWH receiving treatment in Iganga District. [45] The corresponding effective coverage for adults is 10.3% while for the same year a national coverage of 53.5% was reported [14] both based on SPECTRUM software projections to calculate the need for ART. The 2011 national Ugandan HIV survey showed a self reported effective coverage of 50.0% among 1436 respondents who were tested HIV positive and were eligible for ART according to their CD4-count and an effective coverage of 38.2% among 120 respondents from the east central region. [28] To our knowledge the program set up in Iganga has not changed considerably between 2009 and 2011. Therefore regarding the obvious discrepancy in reported coverage we can only assume that in some districts access and retention must be much higher or else, that the assessment to calculate coverage was not as vigorous as in the district we looked at and therefore coverage might have been overestimated. A previous study revealing poor quality of data and care for tuberculosis therapy in several districts in Uganda including Iganga district also casted doubts on national level reports. [46] Further, even if there was a systematic overestimation of ART need as projected by SPECTRUM software, there would still be a striking difference between the district under investigation and the national average. Our results are unlikely to be explained by cohort specific factors since the population appeared similar to previously reported cohorts [7].

While attrition from ART care is one explanation for low effective coverage, some HCs in Iganga also showed low rates of ART initiations per registered PLWH. Other studies have shown that many patients drop out of the programs even before ART initiation with a failure of linkage to ART after HIV testing [47]–[49].

Only 36 children were retained on ART in the district. Eligibility criteria for children vary between programs and thus coverage rates are not easy comparable. Applying the default settings of SPECTRUM resulted in the very low effective coverage of 1.8% for children in this district. A number of interventions such as nutritional support, linkages with associations of PLWH, early infant diagnosis and on-site prevention of mother-to-child transmission (PMTCT) services have been shown to have a favorable impact on pediatric enrollment. [50] Out of these only PMTCT services and engagement of PLWH were routinely available in Iganga district by the time of our study. While the low pediatric coverage is of particular concern it will not be improved unless the overall quality and quantity of services is strengthened.

Comparing the district retention rate with most other reported rates for Uganda, the Iganga District results were lower at the respective time of treatment duration. Reported retention rates range from 0.68 [18] and 0.70 [17], [22] to 0.82 (95% CI 0.78–0.86) [16] at 24 months since ART initiation. Only one other study in a similar public Ugandan setting showed retention rates at a similar level although the definition of LTFU was not completely clear. [24] A Ugandan study from 2002 from one of the first pilot ART programs in SSA showed lower probabilities of retention in care with 0.49 (CI 0.43–0.55) after 12 months [20].

Early warning indicators for ARV drug resistance development include a threshold of less than 20% of ART patients LTFU after 12 months. [51], [52] With a probability of retention in care of 0.72 [CI 0.69 to 0.75] at 12 months after ART initiation, the district misses this target, thus runs a greater risk of drug resistance development at population level. Currently a prevalence of primary drug resistance of 11.6% is reported for Uganda. [11] Further, the development of resistances leads to considerable consequences for the health care system in terms of management of treatment failure and cost for second line treatment [53].

Overall, the differences in retention compared to other studies from Uganda [16]–[18], [22], neighboring countries [54], [55], other countries in SSA [7] or from West Africa [56] may be attributed to either methodological differences in the assessment of retention rates or could suggest that other factors such as external support or home visits influenced better performance. Independent of the cause, the findings of this study call for urgent supportive action such as regular mentoring and supervisory visits which is successfully practiced in Malawi [57] or additional adherence support services. [58] Even lower rates of retention in care after 3 years of ART are described for Lesotho and Mozambique. [59] However, so far not all possible interventions to reduce the rate of patients lost from ART care have been tested, the available studies are few in number and suffer from methodological issues so that more research is needed in this matter [47], [60].

### Differences in Performance between Health Facilities

Despite similar set up and staffing we observed important differences in performance between health facilities with regard to indicators although the same national standards [35] were supposed to be adopted. Our results show that some HCs can perform equally well as a hospital but that others may struggle with retention in care. The finding that HCs had problems with file keeping and documentation in the patient files has been seen in other countries [61] and needs special attention if expansion of the ART program is to reach a sufficient level of quality in terms of monitoring and evaluation. Regardless of other differences, recognition of attrition was poor in all facilities. This is an important observation, as knowing the number of patients alive and attending is crucial, e.g. for drug ordering in the Ugandan pull-system for ARVs [4]. Improvement of recognition of attrition and simplified ways to measure local retention rates as tested in Ethiopia may help health care workers to assess the current situation at their facility [62].

The considerable differences in the distribution of WHO clinical stages between HCs and the hospital may reflect systematic differences in the staging process and its documentation rather than differences in the patient population according to our observation. Striking differences between different clinic sites within the same programme have been described for other countries [40].

We interpret our results such, that HCs can deliver ART and that the differences in performance seem to depend less on structural related factors but rather on clinical staff related factors as absence from duty, motivation or organization of the clinic and some patient related factors. Expert patients may play an important role in reducing the workload of health workers. [63] Interruptions of the supply chain for ARVs as we described it before for Iganga District also led to patient attritions [4] and a study from Mozambique showed that patients attending clinics with higher pharmacy staff burden had a higher risk of attrition [64].

### Risk Factors Associated with Attrition

While gender did not play a significant role, being a young adult or in clinical stages “WHO stage I” or “WHO stage IV” and receiving ART from the hospital were associated with higher risks of attrition. A higher risk of attrition at hospital level has been described before [65] This might partially be explained by the earlier start of the treatment program at the hospital. We suppose that having no symptoms at the last documented visit (“WHO stage I”) might be associated with a higher risk for LTFU as potential adverse effects of ARVs, transport costs and the burden of daily drug intake might outweigh the benefits of ART in this case. Interestingly, a study from Nigeria showed that patients with the highest CD4 counts had also the highest risk for LTFU although clinical staging was not considered in this study [66]. The higher risk associated with WHO stage IV has been described before [8] and may be explained by an increased mortality as shown before for rural Uganda. [67], [24] Finally, young adults (15–24 years) have been seen at higher risk for LTFU also in other studies. [65], [68], [66], [69], [59] Additionally it has been shown that young adults (15–24 years) also have poorer ART treatment outcomes in terms of virological response and virological failure than adults receiving ART [69] and require special attention. So far there is little evidence for improving access and adherence among this vulnerable group [60].

The fact that a longer time span between diagnosis and start of treatment was associated with a lower risk of attrition has to be considered when aiming at earlier start of treatment and at early stages. Our data does not provide an explanation for this observation but concurs with findings from other countries. [68] Counseling strategies will need to be adjusted to address the risk of loss to follow up in early start of treatment. A multi-country study looking at reasons to disengage from ART care proposes that efforts to prevent patients to miss treatment appointments - what may turn into long-term attrition - should always be combined with moves to minimize barriers to re-entry into care to keep attrition to a low level [70].

### Limitations

The approach that we took for this study implies several limitations. The retrospective analysis was deliberately chosen to assess a “real-life” care model outside of prospective research activities or external support, thus our analysis is restricted to the information available at the health facility and district level and remains mainly descriptive and does not allow for example tracing of patients LTFU, analyzing differences in staging procedures or reasons not to start eligible patients at the various health facilities. With regard to the risks for attrition only a randomized controlled trial where all confounding factors are dealt with could prove a causal interferences between attrition and the identified factors we found in our Cox Hazard Model. Further, we looked only at one district reducing the generalizability of our results, although the district seems to represent a fairly average Ugandan district. In any case further research is needed to explore more widely reasons for attrition.

Although every effort was made to retrieve all files and treatment charts, we cannot exclude that some patients whose files were lost or who were labeled LFTU were actually still on treatment. We are however very confident that this number would be low since we were able to visit the facilities at several occasions and actually helped to retrieve files. To seek treatment outside the district or to receive treatment without using the patient chart can be also excluded on a larger scale. Some patients may have transferred themselves to receive treatment from other sources without notification of the system (“silent transfer”) mainly within the district as described before [44] or might have registered twice at different facilities. Two hospital-based studies describe rates of patients LTFU receiving ARV’s from other facilities than the study hospital: up to 31.5% in Uganda [18] and 21% in Malawi [71]. Since we included all ART providing facilities in the district, this rate should be lower here. On the other hand we also assume that all transferred patients continue treatment. This assumption is supported by previous research showing that over 90% of ART patients who transfer-out indeed register with a new site. [72], [73] We found no indication that treatment cards were written without patients actually attending the clinics. It seems therefore unlikely that our data underestimates or overestimates the retention in care to a large extent.

### Conclusions

A very low coverage rate has been revealed in this rural Ugandan district where ART is provided through the public sector falling clearly behind set objectives. Intensified efforts to improve access, linkage to ART, retention in care, and quality of documentation are urgently needed with special attention to children and young adults. Unless these challenges are successfully addressed, the risk of transmission, drug resistance development and mortality is increased. Although even more simplified and community based and supported ART delivery models are needed for the next phase of ART scale up [74], first and foremost strengthening of the existing program is needed including a strengthening of local entities and further integration of HIV care programs into standard Ministry of Health procedures [75], especially before “test and treat” will become a viable strategy in Sub Saharan Africa [47].

## Supporting Information

Table S1 Iganga District HIV projections for 2009.
(DOCX)
