# Supplementary material for: Effective Coverage for Antiretroviral Therapy in a Ugandan District with a Decentralized Model of Care
Source: PLoS One. 2013 Jul 23;8(7):e69433. doi: 10.1371/journal.pone.0069433 (PMC3720624; doi:10.1371/journal.pone.0069433)
Supplement: Table S1 — Iganga District HIV projections for 2009. (DOCX) [file pone.0069433.s001.docx]

**Supplemental Digital Content**

| **Table S 1 Iganga District HIV Projections for 2009** | | | | | | | | |
| --- | --- | --- | --- | --- | --- | --- | --- | --- |
| **Variable** |  | **Age group** | | | | |  | **Sources** |
|  |  | **0-14 years** | **15-49 years** | **50 or older** | **15 or older** | **all ages** |  |  |
|  |  |  |  |  |  |  |  |  |
| **Ratio in Population** | female | 25.96% | 21.67% | 3.57% | 25.25% | 51.21% |  | ***** |
|  | male | 25.33% | 20.41% | 3.05% | 23.46% | 48.79% |  | ***** |
|  | total | 51.30% | 42.08% | 6.62% | 48.70% | 100.00% |  | ***** |
|  |  |  |  |  |  |  |  |  |
| **Inhabitants** | female | 177,097 | 147,819 | 28,089 | 172,200 | 349,297 |  | **†** |
|  | male | 172,800 | 139,198 | 22,039 | 160,003 | 332,803 |  | **†** |
|  | total | 349,898 | 287,017 | 50,128 | 332,202 | 682,100 |  | **†** |
|  |  |  |  |  |  |  |  |  |
| **HIV Prevalence** | female | 1.21% | 7.48% | 1.41% | 6.53% | 3.97% |  | **‡** |
|  | male | 1.22% | 5.20% | 2.34% | 4.83% | 3.05% |  | **‡** |
|  | total | 1.21% | 6.38% | 1.82% | 5.71% | 3.40% |  | **c,†** |
|  |  |  |  |  |  |  |  |  |
| **PLWH** | female | 2,145 | 11,063 | 395 | 11,242 | 13,387 |  | **c,†** |
|  | male | 2,101 | 7,239 | 515 | 7,733 | 9,834 |  | **c,†** |
|  | total | 4,246 | 18,302 | 910 | 18,975 | 23,221 |  | **c,†** |
|  |  |  |  |  |  |  |  |  |
| **ART need per 10.000 inhabitants according to national guidelines** | female | 57.9 | 211.7 | 97.3 | 193.7 | 128.5 |  | **‡** |
|  | male | 58.2 | 156.5 | 164.0 | 157.5 | 108.5 |  | **‡** |
|  |  |  |  |  |  |  |  |  |
| **Need for ART according to national guidelines** | female | 1,025 | 3,129 | 273 | 3,335 | 4,360 |  | **†,‡** |
|  | male | 1,005 | 2,179 | 361 | 2,520 | 3,525 |  | **†,‡** |
|  | total | 2,030 | 5,308 | 634 | 5,855 | 7,885 |  | **†,‡** |
|  |  |  |  |  |  |  |  |  |
| **ART need per 10.000 inhabitants using a CD4 threshold for eligibility of 350/µl in 2009** | female | 57.9 | 283.6 | 106.3 | 255.7 | 160.7 |  | **c** |
|  | male | 58.2 | 205.0 | 178.8 | 201.7 | 130.9 |  | **c** |
|  |  |  |  |  |  |  |  |  |
| **Need for ART using a CD4 threshold for eligibility of 350/µl in 2009** | female | 1,026 | 4,192 | 299 | 4,404 | 5,430 |  | **†,‡** |
|  | male | 1,005 | 2,854 | 394 | 3,227 | 4,232 |  | **†,‡** |
|  | total | 2,031 | 7,046 | 693 | 7,631 | 9,662 |  | **†,‡** |
|  |  |  |  |  |  |  |  |  |
| *****: Data from Iganga-Mayuge Health and Demographic Surveillance System (HDSS) sentinel survey in 2006 including 51,114 inhabitants of the Iganga part of the HDSS | | | | | | | | |
| **†**: Projections calculated using the 2009 district population projection of the Ugandan Bureau of Statistics (14) and the sex- and age group-ratios according to HDSS sentinel survey (*) | | | | | | | | |
| **‡**: Projections calculated using SPECTRUM Version 4.43 beta 7 with default settings for Uganda, medium total fertility rate assumptions (5,7) and medium life expectancy assumptions (59,7 years). | | | | | | | | |
